# Correlation Between Optic Nerve Sheath Diameter at Initial Head CT and the Rotterdam CT Score

**DOI:** 10.7759/cureus.41995

**Published:** 2023-07-17

**Authors:** Aletor O Amakhian, Elohor B Obi-Egbedi-Ejakpovi, Eghosa Morgan, Ademola A Adeyekun, Munir M Abubakar

**Affiliations:** 1 Radiology, Sandwell and West Birmingham NHS (National Health Service) Trust, Birmingham, GBR; 2 Radiology, Irrua Specialist Teaching Hospital, Irrua, NGA; 3 Neurosurgery, Irrua Specialist Teaching Hospital, Irrua, NGA; 4 Radiology, University of Benin Teaching Hospital, Benin, NGA

**Keywords:** neuro-critical care, noninvasive intracranial pressure monitoring, midline shift, rotterdam computed tomography score, severity, optic nerve sheath diameter, traumatic brain injury

## Abstract

Introduction

Intracranial findings on imaging have long been used in assessing the severity of traumatic brain injury (TBI); the Rotterdam CT scoring (RCTS) is a more recent tool. Estimating the optic nerve sheath diameter (ONSD) at computed tomography (CT) can be another valuable predictor of the severity of the injury, especially as both ONSD and the RCTS are proven to be independent predictors of raised intracranial pressure (ICP). The study objective was to determine the correlation between ONSD at initial head CT and RCTS.

Material and methods

We observed 40 consecutive confirmed TBI cases at their initial head CT examinations in the emergency department for ONSD and the presence of other intracranial findings necessary to derive RCTS. The data were prospectively collected and analyzed, with statistical significance set at p ≤0.05 at 95% CI.

Results

The mean ONSD positively correlated with the Rotterdam CT score (r=0.368, p=0.019). A cut-off value of 6.83 mm was extrapolated from the receiver operator characteristic (ROC) curve as the mean binocular ONSD that best predicted severe RCTS (≥4) (sensitivity: 73.3%, specificity: 80%, positive predictive value: 68.7%, negative predictive value: 83.3%). The area under the curve (AUC) was 0.780 (p=0.003). Binary logistic regression analysis revealed an odd ratio (OR) of 11.000 (95% CI: 2.438-49.627; p=0.002).

Conclusion

TBI patients with high RCTS have wide mean binocular ONSD. Those with average binocular ONSD above the cut-off value are likelier to have severe TBI. With the documented good correlation, ONSD may become very useful in informing the clinical decision for sequential CT scans in TBI patients and, therefore, reducing the cumulative radiation burden from needless exposures. Furthermore, the non-invasive nature of its assessment will have more clinical relevance in resource-limited settings, where the skills and equipment for ICP monitoring are either not readily available or too expensive to be used routinely.

## Introduction

Traumatic brain injury (TBI) is a common occurrence following high-impact trauma. It is defined as the alteration of brain function or other evidence of brain pathology caused by an external mechanical force [1]. In Nigeria and other developing countries, road traffic accidents (RTA) account for a more significant proportion of the mechanisms of injury [1-3]. TBI is a significant cause of morbidity and mortality and requires timely intervention. The severity of each case usually guides this.

The classic clinical features may include progressive deterioration of consciousness, headache, and vomiting, among other signs. Imaging findings include soft tissue injury, skull fractures, contusions, intra- and extra-axial haemorrhages, and cerebral oedema, leading to increased intracranial pressure (ICP). Depending on various factors, this may be evident by the shift of midline brain structures, cisternae compression, and sulcal effacement [4]. Great efforts by Marshall et al. [5] in 1991 and, most recently, Maas et al. [6] in 2007, who proposed the Rotterdam computed tomography scoring (RCTS), have classified TBI based on CT findings. The latter was more encompassing and addressed some shortcomings of the former, taking into account the presence of intraventricular, epidural, and subarachnoid haemorrhage (SAH) in addition to the presence of midline shift and basal cistern appearance [5-6]. More recent research has demonstrated high RCTS correlating well with a higher likelihood of elevated ICP especially given a midline shift of above 5 mm [7]. It has, over time, been validated by the outcome of such studies to be good prognosticators in TBI [8-9].

Several neuroimaging efforts have targeted novel methods for triaging head injury and early prognostication. The most popular, which is the ONSD measurement, has become a valuable adjunct to other clinical and laboratory prognostic indices, such as the Glasgow Coma Score (GCS), Glasgow Outcome Score (GOS), lumbar puncture manometry, and leucocytosis [1]. It has found usefulness, particularly when elevated ICP is suspected, but an invasive method is not feasible [9-14]. Several studies have validated the ONSD's correlation with elevated intracranial pressure [10,12,15-17].

Since both the brain injury classification and optic nerve sheath assessment can reasonably predict the likelihood of elevated ICP and the outcome of TBI, there is a need to correlate these independent variables [8-9]. Although some studies have tried to find the correlation between the ONSD and the Marshall Classification system, only a few documented studies that tried to establish its correlation with the newer Rotterdam scoring system exist in the literature [9,12]. To the best of our knowledge, there are no universally accepted standards for the applicability of ONSD. Therefore, this study aimed to determine the correlation between ONSD and the RCTS at early CT of TBIs and add to the existing body of knowledge in furtherance of standardization.

## Materials and methods

This prospective observational study was conducted over six months, from August 2020 to January 2021, at the Radiology Department in Irrua, Nigeria. Irrua Specialist Teaching Hospital is a rural tertiary centre with trauma and neurosurgical units. Forty consecutive confirmed cases of TBI referred for CT examination were recruited regardless of gender. Patients below 18 years of age, those with a penetrating head injury, direct orbital/eye trauma, a pre-existing ocular disease affecting the optic nerve and orbital cavity, and pre-existing systemic conditions with orbital manifestation were excluded.

Based on their presenting GCS, they were stratified into three groups (mild, moderate, and severe). Secondary data like age, gender, mode of injury, and date of admission were retrieved from the patient’s medical record as documented by the managing neurosurgeon. The patients were anonymised during the analysis. Approval was obtained from the Research and Ethics Committee of the Irrua Specialist Teaching Hospital (ISTH/HREC/20192211/052). Informed written consent was obtained before the recruitment of subjects, either directly from the patients or their caregivers for those who were unconscious.

The technique for evaluation of ONSD at computed tomography

CT examinations were done with a Neuviz-16 multi-slice scanner (Philips and Neusoft Medical Systems Company Ltd., China; 2013). Patients were placed supine, and appropriate restrainers with head stabilizers were in place to prevent involuntary motion. As part of a standard trauma CT scan protocol, 3 mm unenhanced axial head CT slices were acquired from the base of the skull to the vertex. All scans were done in the horizontal plane, with the gantry at zero degree. The ONSD was measured at the admission head CT on each side using the axial slice on a soft tissue window (Hounsfield unit range +25 to +50). Measurements were done on a DICOM viewer (64 bits) software, using electronic callipers, 3 mm immediately behind the sclera, at 90 degrees to the long axis of the optic nerve, as a section through its centre. The diameters measured for the patient’s left and right eyes were averaged to yield the mean value [14-18].

Radiological interpretation of the cranial CT findings

The researchers were blinded to the patient’s medical history, circumstances of the TBI, and their respective severity scores. The presence of intracerebral, subarachnoid, intraventricular haemorrhage, basal cistern compression, cortical sulcus effacement, or midline shift of more than 5 mm on the initial CT scan was documented. According to the RCTS system described by Maas et al. [6] (Table 1), each patient’s initial head CT scan was scored on a different occasion to reduce bias further. They were then divided into two groups: mild (RCTS score of 2 and 3) and severe (RCTS ≥4).

**Table 1:** Rotterdam CT scoring system. In this classification, one extra point (+1) is added to the sum score to make it numerically a total of six (6) points, consistent with the motor score of both the Glasgow Coma Scale and the Marshall classification.

Predictor	Score
Basal cistern	
Normal	0
Compressed	1
Absent	2
Midline shift	
No shift or shift ≤ 5 mm	0
Shift > 5 mm	1
Epidural mass lesion	
Present	0
Absent	1
Intraventricular blood or subarachnoid haemorrhage	
Absent	0
Present	1
Sum score	+1

Method of data analysis

The data was entered into a spreadsheet and analyzed using SPSS (IBM Corp. Released 2012. IBM SPSS Statistics for Windows, Version 21.0. Armonk, NY: IBM Corp). Summary statistics for quantitative variables were expressed as mean ± standard deviation. Categorical parameters, defined as frequency, were compared by the chi-square test. Pearson’s correlation and a regression model were also used to ascertain relationship differences. The receiver operator characteristic (ROC) curve was obtained from sensitivity and specificity data to determine the cut-off value of ONSD correlating with the RCTS. The cut-off value was determined graphically, and after that, the positive and negative predictive values and the odd ratio were calculated with 95% confidence intervals (CIs). p ≤0.05 was set to consider the statistical significance.

## Results

**Figure 1:**
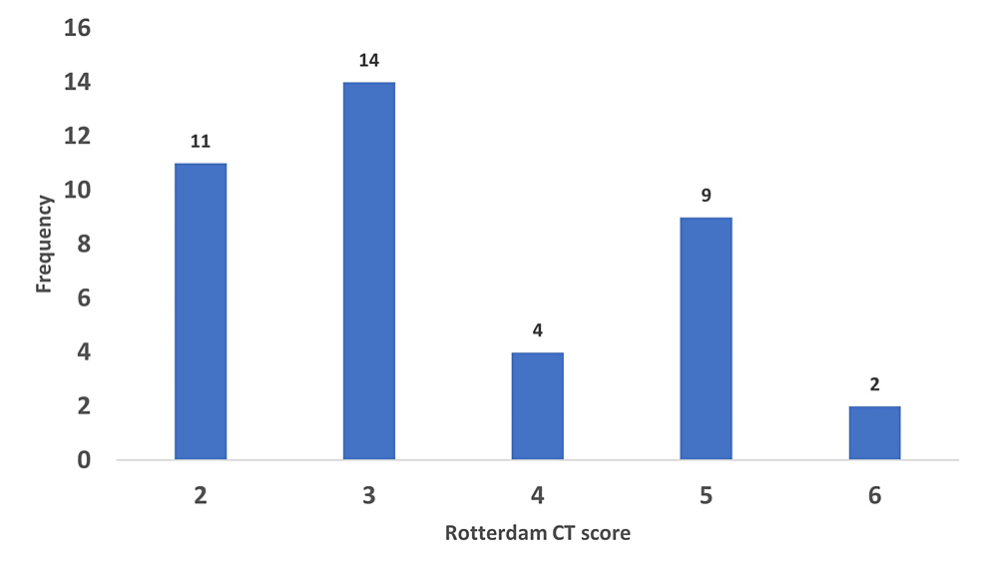
Rotterdam CT score of the cases.

Forty subjects were studied, out of which eight (20.0%) had a severe brain injury as assessed by their GCS at presentation. RTA was the most common mechanism of injury at 62.5% (Table 2). Figure 1 shows the distribution of cases among the various RCTS classes.

**Table 2:** Mode of injury and presenting GCS of the 40 cases. GCS: Glasgow Coma Score, RTA: road traffic accidents.

Variable	Frequency (n)	Percentage (%)
Mechanism of injury		
RTA	25	62.5
Assault	9	22.5
Fall	6	15.0
GCS at presentation		
Mild brain injury	20	50.0
Moderate brain injury	12	30.0
Severe brain injury	8	20.0

**Figure 2:**
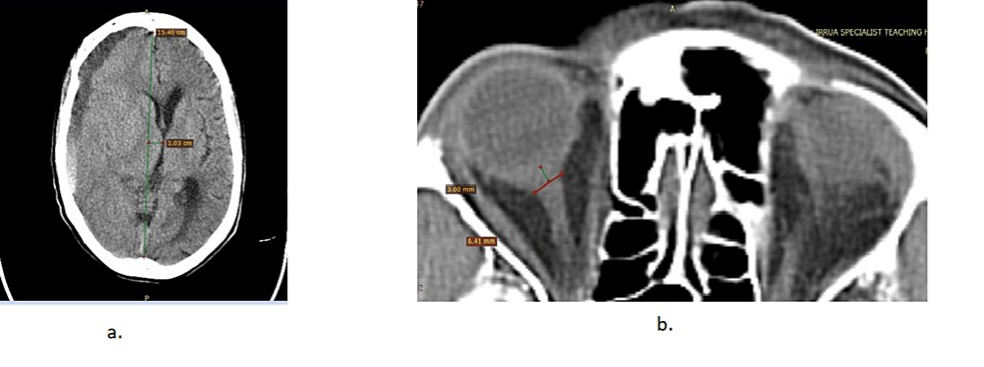
Non-enhanced CT images of the head. (a) There is an extensive crescentic lesion in the right parietal convexity, with a fluid-fluid (haematocrit) level. It has caused marked effacement of the regional sulci and the anterior horn of the right lateral ventricle, with significant contralateral shift (>5 mm) of the midline brain structures. (b) At the level of the orbit, there is increased ONSD (6.41 mm) taken 3 mm behind the globe. Only the right intraconal ONSD complex is fully shown in this section. ONSD: optic nerve sheath diameter.

Using the RCTS, 15 of the 40 subjects (37.5%) were classified as severe brain injury (RCTS ≥ 4), while 25 (62.5%) had a mild brain injury (Table 3). In terms of the occurrence of CT findings among the cases (Table 3), normal basal cistern morphology was observed in seven patients (17.5%), compressed basal cisterns in 23 (57.5%), and absent basal cisterns in the remaining 10 (25.0%). It was observed that 27 (67.5%) cases had a midline shift of < 5 mm, whereas 13 (32.5%) had a shift of ≥ 5 mm. Whereas 13 (32.5%) cases had epidural haematoma, no epidural haematoma was demonstrable in 27 (67.5%). Only 17 (42.5%) had intracerebral haemorrhage/contusion, while 23 (57.5%) did not. While 14 (35.0%) had either subarachnoid or intraventricular haemorrhage, 26 (65.0%) had no such findings. Figure 2 shows selected images of one of the cases, with a widened ONSD, and some of the findings mentioned above.

**Table 3:** CT findings among the cases.

Variable	Frequency (n)	Percentage (%)	
Intracerebral haemorrhage/contusion			
Present	17	42.5	
Absent	23	57.5	
Intraventricular/subarachnoid haemorrhage			
Present	14	35.0	
Absent	26	65.0	
Epidural mass lesion			
Present	13	32.5	
Absent	27	67.5	
Basal cisterns			
Normal	7	17.5	
Compressed	23	57.5	
Absent	10	25.0	
Midline shift			
≤ 5 mm	27	67.5	
> 5 mm	13	32.5	
Rotterdam CT score			
Mild	25	62.5	
Severe	15	37.5	

ONSD was positively correlated with RCTS for both the right and left eyes (r of 0.514 and 0.132 for the right and left eyes, respectively). It was observed that an increase in the average binocular ONSD corresponded to a higher value of the RCTS (r=0.368); this was statistically significant (p=0.019) (Table 4).

**Table 4:** Correlation between mean binocular ONSD and RCTS. R: correlation coefficient, r^2^: coefficient of determination, ONSD: optic nerve sheath diameter, RCTS: Rotterdam CT score, LE: left eye, RE: right eye.

Variable	RCTS		
	R	r^2^	p-value
RE ONSD	0.514	0.651	0.001
LE ONSD	0.132	0.897	0.418
Average binocular ONSD	0.368	0.383	0.019

**Figure 3:**
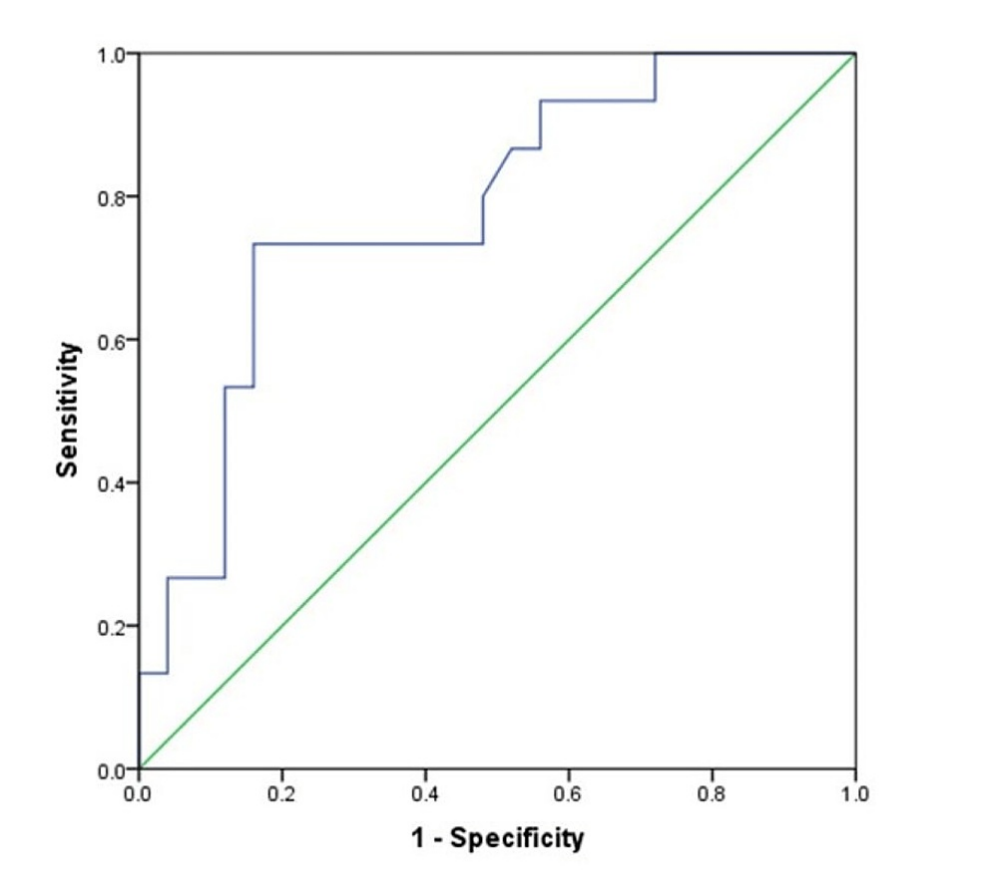
Receiver operator characteristic curve: ONSD as a predictor of RCTS. ONSD: optic nerve sheath diameter, RCTS: Rotterdam CT score.

Figure 3 shows that the area under the average binocular ONSD ROC curve at 0.780 (95% CI: 0.632-0.928, p=0.003) is a good predictor of severe RCTS. From this curve, a cut-off ONSD value of 6.83 mm was extrapolated, equal to or above which predicted a severe RCTS (sensitivity: 73.3%, specificity: 80%), with a positive predictive value (PPV) of 68.7%, negative predictive value (NPV) of 83.3%, and odds ratio (OR) of 11.000 (95% CI: 2.438-49.627; p=0.002) (Table 5).

**Table 5:** Average binocular ONSD and RCTS of cases. PPV (68.7%); NPV (83.3%). NSD: optic nerve sheath diameter, RCTS: Rotterdam CT score, PPV: positive predictive value, NPV: negative predictive value.

Variable		RCTS				ꭓ^2^	p-value	
		Mild (n=25) (%)		Severe (n=15) (%)				
Average binocular ONSD (mm)								
< 6.830	20 (80.0)		4 (26.7)		11.111		0.001	
≥ 6.830	5 (20.0)		11 (73.3)					

## Discussion

The optic nerve is invested in a sheath comprising the three meningeal coverings, which form the optic nerve sheath complex. Direct communication has been documented between the subarachnoid space of the optic nerve and the chiasmal cistern of the brain, providing a homogenous CSF pressure between the two subarachnoid compartments so that any gradients are attenuated through respective CSF transfers [18]. An increase in ICP would cause CSF to be transmitted through this connection and manifest as a widening of the ONSD measurable at the intraconal portion.

In this study, wider ONSD was associated with higher RCTS, which was found to be statistically significant (p=0.019). This agreed with the analysis by Das et al. [7], who noted that worsening Rotterdam scores were consistent with an incremental increase in ONSD. It was also in agreement with the work by Kayadibi et al. on a paediatric population, where they found a positive correlation between ONSD and high RCTS for paediatric patients [19]. Using ultrasonography (USG), Thotakura et al. equally demonstrated a positive correlation between ONSD and radiological scores (Marshall and Rotterdam scores) [20]. Furthermore, we observed a similar positive correlation of RCTS versus the ONSD for the respective right and left eyes, and this compared well with the findings from a retrospective cohort study by Majeed et al. [16], where it was documented that the effects of midline shift and laterality of lesions did not affect the correlation.

We went on to demonstrate that cases with non-critical RCTS, regarded as mild TBI, had average binocular ONSD values of 6.17 to 6.70 mm with a mean of 6.50 ± 0.47 mm. This was in accordance with the findings by Bekerman et al. [21], who reported that in a majority (82%) of cases of TBI without haemorrhage (n=591), the ONSD is significantly enlarged, indicating elevated ICP even if CT scans are negative. Their study documented enlarged right/left ONSDs as 6.7 ± 1.0/6.7 ± 0.9 mm, respectively. These values, like ours, are noticeably above the range for normal controls reported in some literature. Aduayi et al. [15] in Ife (Southwest Nigeria) documented a similar pattern, although they had employed USG for their work; hence, our outcomes may not be comparable. A probable implication of this observation is that those with mild TBI may already have cerebral blood volume and perfusion changes, enough to distort the subarachnoid sleeve around the optic nerve, albeit minimally. However, this disagreed with the findings by Das et al. [7], where the mean ONSD of subjects in their mild TBI groups was within the normal range for controls. They had documented mean ONSD for RCTS 2 and RCTS 3 as 3.3 ± 0.39 mm and 4.1 mm ± 0.05 mm, respectively, while that for RCTS ≥ 4 (severe TBI) was 4.83 mm ± 0.40 mm. Their mean value correlating with severe TBI was also noted to be relatively lower than ours (7.04 mm ± 0.52) and that of Vaiman et al. [22], who indicated that ONSD was enlarged to 6.6 ± 0.8 mm in 95% of patients with intracerebral haemorrhage or SAH. These dissimilarities may be attributable to measurement bias and the patient selection method, especially with the work by Das et al. [7] being retrospective.

The higher the average binocular ONSD, the greater the severity of TBI given by a critical RCTS (≥4). An average binocular ONSD ≥ 6.83 mm was found to be indicative, with a PPV of 68.7% and an NPV of 83.3%, of severe TBI. An odds ratio of about 11.1 revealed that those with average binocular ONSD ≥6.83 mm were about 11 times more likely to have severe TBI than those with values <6.83 mm. The area under the ROC curve of 0.780 indicated that average binocular ONSD is a good test for assessing the severity of injury in cases of TBI (sensitivity: 73.3%, specificity: 80.0%). These patterns aligned with the works by Das et al. [7], which documented 0.914 for the area under the curve (95% CI: 0.907-0.974), and Luyt et al. [17]. Although these latter two works documented higher values for some of these parameters, they were consistent with our findings. The discrepancies could have resulted from different protocols employed by the researchers in their measurements and study population differences.

Fujimoto et al. [23] observed that the preoperative RCTS was more significantly associated with unfavourable outcomes (OR=15.29, 95% CI: 2.50-93.53, p=0.003) than the initial RCTS (OR=3.66, 95% CI: 1.29-10.39, p=0.02). They concluded from their work that assessing changes in RCTS over time may serve as a prognostic indicator in TBI and can help determine which patients require decompressive craniectomy. Similar research by Shetty et al. [24] established a correlation between the Rotterdam scores, the need for sequential CTs, and the cumulative radiation dose, which according to them, had helped develop a preliminary protocol that could be followed to bring about better planned and efficient patient care. They opined that there is no additional role of sequential CT for the cases with a Rotterdam score of 1 or 2 in the initial CT unless there is clinical evidence of deterioration. They noted that Rotterdam score 3 needs sequential CT after 24 hours, and Rotterdam scores 4 and 5 need sequential CT after 12 hours if surgical intervention is delayed. In light of the above and the additional evidence from our study, the applicability of ONSD measurement in the dynamic monitoring of RCTS can, therefore, not be overemphasized in case management, as it would allow for rapid assessment at the ED.

Limitations

There are several limitations to our study. One is that the principal investigator performed all measurements; hence, the intra-observer variability could not be assessed. However, an average of three readings was taken to minimize this effect. The small sample size and the lack of literature on this subject made adequate comparisons difficult. Despite the limitations, our study will provide helpful information and context for more multi-centre studies with larger sample sizes, in which these findings could be extrapolated.

## Conclusions

TBI patients with high RCTS have wide mean binocular ONSD. Those with average binocular ONSD above the cut-off value are likelier to have severe TBI. With the documented good correlation, ONSD may become very useful in informing the clinical decision for sequential CT scans in TBI patients and, therefore, reducing the cumulative radiation burden from needless exposures. Furthermore, the non-invasive nature of its assessment will have more clinical relevance in resource-limited settings, where the skills and equipment for ICP monitoring are either not readily available or too expensive to be used routinely.

We propose that ONSD should be documented in the initial CT reports of TBIs. Also, knowing that USG values are equally representative, clinicians can readily afford sequential ONSD monitoring using bedside USG as an alternative way of assessing changes in ICP and corresponding RCTS from the baseline. This could warrant and justify re-exposing the patient to CT examination.
